# Subtypes of breast cancer show different spatial distributions of brain metastases

**DOI:** 10.1371/journal.pone.0188542

**Published:** 2017-11-20

**Authors:** Sunghyon Kyeong, Yoon Jin Cha, Sung Gwe Ahn, Sang Hyun Suh, Eun Ju Son, Sung Jun Ahn

**Affiliations:** 1 Severance Biomedical Science Institute, Yonsei University College of Medicine, Seoul, Korea; 2 Department of Pathology, Gangnam Severance Hospital, Yonsei University, College of Medicine, Seoul, Korea; 3 Department of Surgery, Gangnam Severance Hospital, Yonsei University, College of Medicine, Seoul, Korea; 4 Department of Radiology, Gangnam Severance Hospital, Yonsei University, College of Medicine, Seoul, Korea; University of North Carolina at Chapel Hill School of Medicine, UNITED STATES

## Abstract

The aim of our study was to test the hypothesis that the spatial distribution of breast cancer brain metastases (BM) differ according to their biological subtypes. MR images of 100 patients with BM from primary breast cancer were retrospectively reviewed. Patients were divided according to the biological subtype of the primary tumor, (triple-negative: 24, HER2 positive: 48, luminal: 28). All images marked with BMs were standardized to the human brain MRI atlas provided by the Montreal Neurological Institute 152 database. Distribution pattern of BM was evaluated with intra-group and intergroup analysis. In intra-group analysis, hot spots of metastases from triple-negative are evenly distributed in the brain, meanwhile BMs from HER2 positive and luminal type occur dominantly in occipital lobe and cerebellum. In intergroup analysis, BMs from triple-negative type occurred more often in frontal lobe, limbic region, and parietal lobe, compared with other types (*P* < .05). Breast cancer subtypes tend to demonstrate different spatial distributions of their BMs. These findings may have direct implications for dose modulation in prophylactic irradiation as well as for differential diagnoses. Thus, this result should be validated in future study with a larger population.

## Introduction

Brain metastases (BMs) are the most commonly encountered malignant tumors occurring in the CNS, outnumbering primary CNS tumors by more than 10-fold[1]. Breast cancer is the second most frequent cause of BM after lung cancer, with metastases occurring in 10–16% of patients[2]. Median survival ranges from 3 to 15 months following metastatic spread to the brain, making BMs one of the major causes of systemic cancer-related mortality[3]. Recently, the incidence of BMs has increased because of improvements in treatment for primary cancers and more advanced imaging techniques[4,5].

Breast cancer can be divided into several biologic subtypes on the basis of their clinical, histopathological, and molecular features. Further, breast cancer can be classified on the basis of their gene expression profiles into luminal, basal, and HER2-positive, with each subtype showing a clearly different prognostic significance[6,7]. The subgroups of patients with triple-negative and human epidermal growth factor receptor 2 (HER2)-positive breast cancer are at a higher risk for development of BMs[8–10]. The onset of BMs in triple receptor-negative breast cancer is earlier than that observed in other subtypes, and the overall survival rate is particularly poor, when compared to other subtypes[11].

Treatment options for patients with breast cancer BMs are limited and include surgical resection, whole-brain radiation therapy, stereotactic radiosurgery, chemotherapy, and targeted therapy[12–14]. Prophylactic cranial irradiation improves the survival rate of patients with lung cancer BMs[15,16], and may represent a novel approach for select patients with breast cancer BMs[17]. Thus, understanding the spatial distributions of BMs by breast cancer subtype may allow for more precise prophylactic irradiation adjustments and could lead to the development of novel targeted therapy.

Biological characteristics of tumors could affect the spatial distribution of their BMs. For example, the probability of cerebellar metastases is higher in lung and breast cancer[18]. BMs are typically located in watershed areas such as the gray-white matter junction[19]. Recently, Takano et al. reported that lung cancer BMs with an epidermal growth factor receptor (EGFR) L858R mutation occurred more often in the caudate nucleus, cerebellum, and temporal lobe than those with an EGFR exon 19 deletion[20]. We hypothesized that breast cancer BMs have different spatial distributions according to their biological subtypes.

## Materials and methods

### Participants

**Fig 1:**
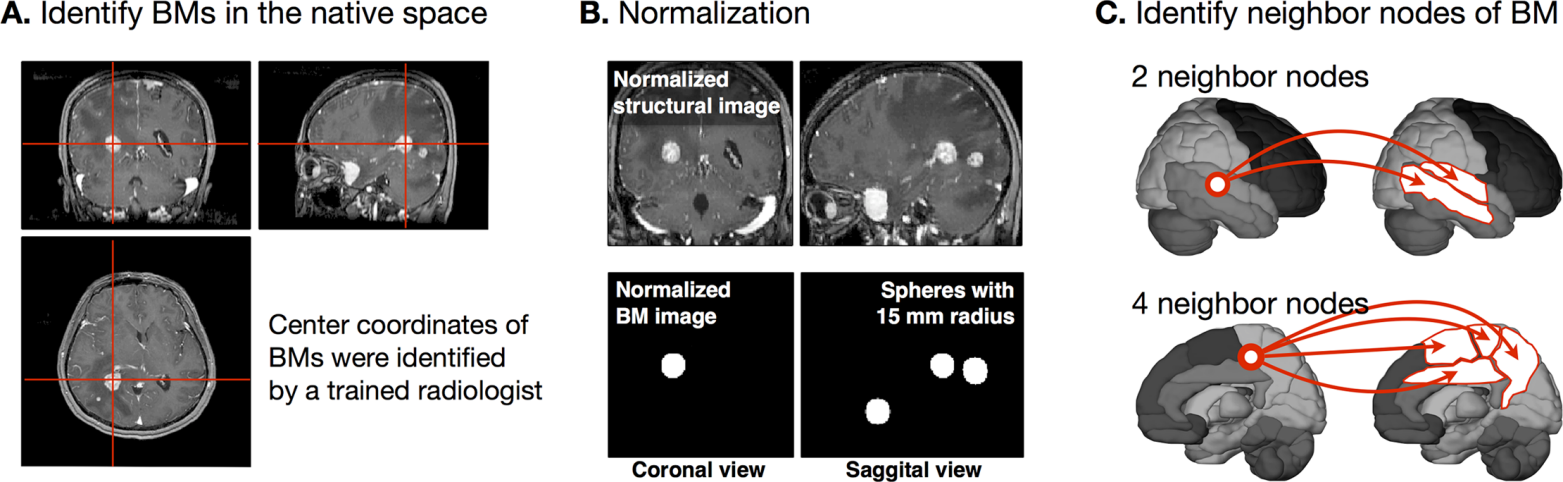
Flowchart for mapping brain metastases (BMs) in the standardized automated anatomical labeling (AAL) template space. The center coordinates of BMs in the native space were identified by a trained radiologist (A). The individual center coordinates of BMs were normalized in the Montreal Neurological Institute space and normalized lesion maps were extended to a site-centered spherical shape with a radius of 15 mm (B). The AAL template was matched with the spherical BMs in the MNI space. We assigned 1 if there were overlaps between BM and AAL nodes (C). For examples, 1) if the center coordinate of BM is located around the temporal regions and its spherical ROI with 15 mm radius were overlapped with the superior temporal gyrus and middle temporal gyrus, then those two temporal gyri were identified as neighbor nodes (top image of Fig 1C). 2) if the center coordinate of BM is located around the paracentral lobule and the middle cingulate gyrus and its spherical ROI with 15 mm radius were overlapped with the middle cingulate gyrus, paracentral lobule, supplementary motor area, and precuneus, then these four medial regions were identified as neighbor nodes (bottom image of Fig 1C).

We retrospectively reviewed data for breast cancer patients with BM who underwent gadolinium-enhanced brain MRI from 2009 to 2016. A total of 128 patients were identified. Of theses 27 patients were excluded for the following reasons (Fig 1): (1) previous neurosurgery or brain radiation therapy (n = 10); (2) presence of other malignant disease (n = 5); and (3) absence of the immunohistochemistry profile of breast cancer (n = 12). A total of 101 patients was remained after selection criteria. However, an unknown error occurred during exporting from the hospital database to the local computer in one of 101 brain MRIs, and this case was removed in the statistical mapping. Finally, gadolinium-enhanced 3D T1WIs of 100 breast cancer patients in whom BMs were initially diagnosed were included in this analysis (slice thickness <1.5 mm on 3.0T MRI). Patients were divided into three groups according to biological subtypes on the basis of the expression of estrogen (ER), progesterone (PgR), and HER2. Immunohistochemistry was carried out for the evaluation of the level of ER, PgR, and HER2 expression of primary breast cancer. Florescence in-situ hybridization analysis of HER2 amplification was carried out in immunohistochemistry 2+ cases. The three subtypes were triple negative (ER-, PgR-, HER2-), HER2 positive (HER2(+), any ER/PgR), and luminal (ER/PgR(+), HER2(-)). The current study design and use of clinical data was approved by the institutional review board of Gangnam Severance hospital The requirement to obtain informed consent was waived, and all data were fully anonymized.

### Image registration and frequency map reconstruction

DICOM gadolinium-enhanced 3D T1WIs were reviewed by Two radiologist (A.S.J, S.S.H) to identify the focus of the brain metastases. They independently marked brain metastases and recorded coordinates (x,y,z) of brain metastases. Interobserver agreement was assessed using the Concordance Correlation Coefficient (CCC). The coordinates of reader 1 was used for further analysis. Fig 1 presents the flowchart for mapping brain metastases (BMs) in the standardized automated anatomical labeling (AAL) template space[21]. The primary sites of occurrence were then individually mapped in Neuroimaging Informatics Technology Initiative format by assigning ones for the manually identified lesions and zeros otherwise (Fig 1A). After converting DICOM images to the Neuroimaging Informatics Technology Initiative format using the dcm2nii software (http://cabiatl.com/mricro/mricron/dcm2nii.html), the images were normalized to the standard space using the Statistical Parametric Mapping (SPM12; http://www.fil.ion.ucl.ac.uk/spm/software/spm12) software. Individually coregistered images of the BMs and a manually marked lesion map were standardized to the human brain MRI atlas provided by the Montreal Neurological Institute (MNI) 152 database with a 1×1×1 mm voxel size. For frequency map reconstruction, normalized lesion maps were extended to a site-centered spherical shape with a radius of 15 mm (Fig 1B). Subsequently, these spherical BMs were matched with the AAL template. Initially, we assigned zeros for 116 cortical, subcortical, and cerebellar regions in AAL template space, we then assigned ones if there were overlaps between individual spherical BMs and AAL regions. Finally, all BM heat maps were reconstructed and superimposed in the AAL space to determine the frequency of metastasis occurrence (Fig 1C).

### Analysis of spatial distribution of brain metastasis

For intra-group analysis, heat map is generated based on the percentage of brain metastasis involving specific AAL region, which was defined as number of patients whose brain metastasis involving specific AAL region per total number of patients with a specific breast cancer subtype. Top10% of ALL regions in frequency of metastases could be listed using heat map. For intergroup analysis, the frequency of occurrence of metastasis was compared between biological subtypes of the breast cancer: (1) triple-negative or non-triple-negative; (2) HER2 or non-HER2; and (3) luminal or non-luminal. Single-subject BM maps were entered into a second-level analysis using a χ2 test crosstab analysis to assess group level significance. The significance threshold was set at P < .05

## Results

In total, 100 patients were analyzed: 24 patients had triple-negative breast cancer, 48 patients had HER2-positive breast cancer, and 28 patients had luminal breast cancer. All patients were female. Age at initial diagnosis of breast cancer was not significantly different among breast cancer subtypes (46.45 ± 10.88 for triple-negative, 49.5 ± 11.45 for HER2, 46.75 ± 9.73 for luminal; *P* = 0.26). Number of brain metastases per patient did not significantly differ by subtype (5.33 ± 5.78 for triple-negative, 4.71 ± 6.58 for HER2, 5.35 ± 6.69 for luminal; *P* = 0.88). However, triple-negative and HER2-positive breast cancer showed a shorter time interval until the onset of BMs than luminal breast cancer (23.5 ± 23.36 months for triple-negative, 19 ± 29.54 months for the HER2 subtype, and 42 ± 45.51 months for luminal subtype; *P* < .01, Table 1)

**Table 1:** Patient characteristics.

	All cancers	Triple negative (ER-, PgR, HER2-)	HER2 (HER2+, any ER/PgR)	Luminal (ER/PgR+, HER2-)	P-value
**Patients (n)**	100	24	48	28	
**Female (n)**	100	24	48	28	
**Age (year) at diagnosis of primary tumor**	47 ± 10.91	46.45 ± 10.88	49.5 ± 11.45	46.75 ± 9.73	.26
**Duration between primary tumor and brain metastases (months)**	22 ± 35.01	23.5 ± 23.36	19 ± 29.54	42 ± 45.51	< .01
**Number of brain metastases per patient**	5.04 ± 6.37	5.33 ± 5.78	4.71 ± 6.58	5.35 ± 6.69	.88

### Interobserver agreement

The coordinates of brain metastases had good reproducibility between two radiologists. Their Concordance Correlation Coefficient was 0.996 (0.994–0.997)

### Frequency map of brain metastases in each subtype (intra-group analysis)

**Fig 2:**
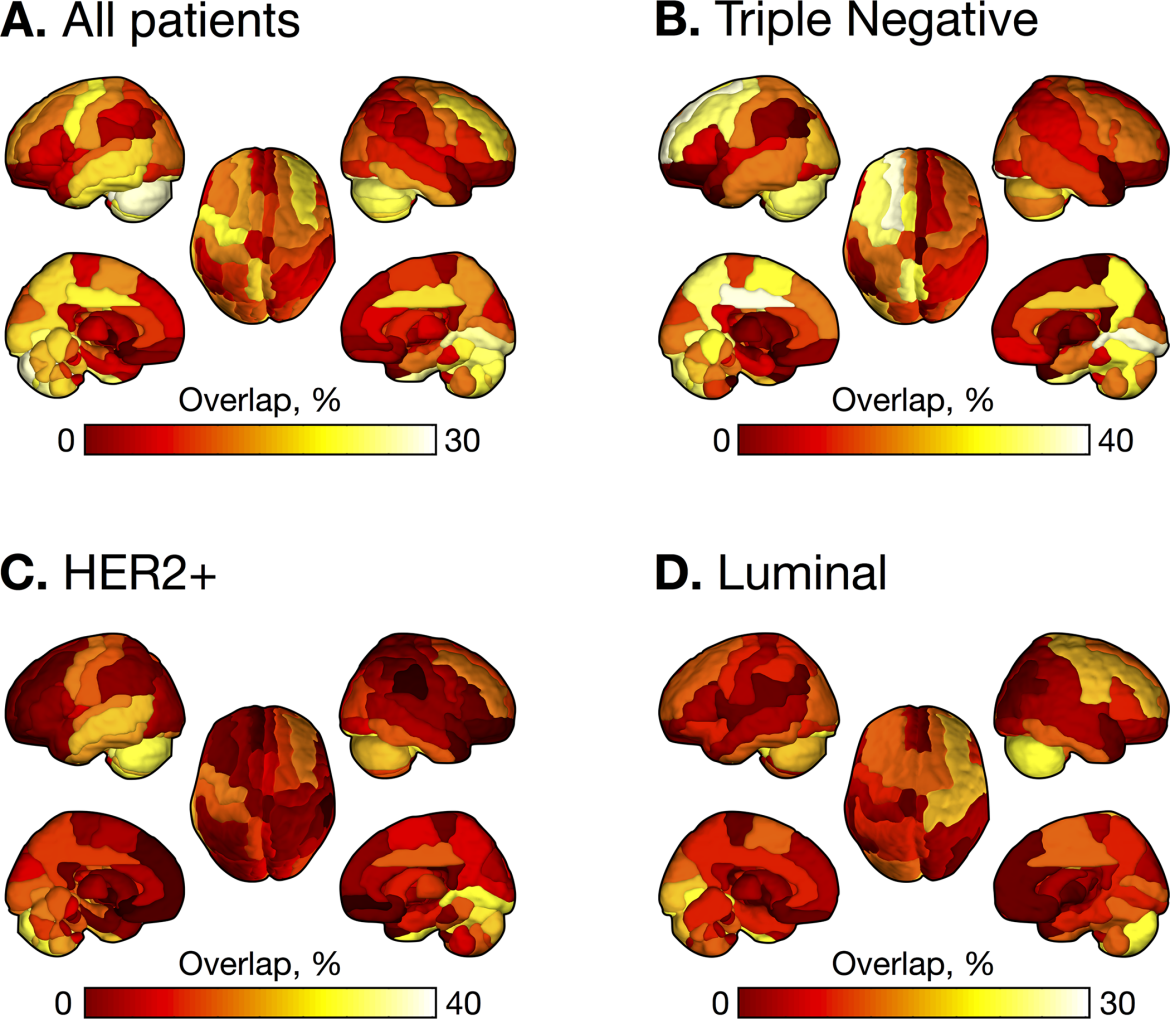
Heat maps of brain metastases (BMs). Overlapping of BMs across all patients with breast cancer (A), patients with triple negative type (B), patients with HER2+ type (C), and patients with Luminal type (D). Color-bars indicate the percentage of BMs. Abbreviations: CBL, cerebellum; IFG, inferior frontal gyrus; MFG, middle frontal gyrus; L, left; R, right; MO, middle occipital; PMC, premotor cortex.

**Fig 3:**
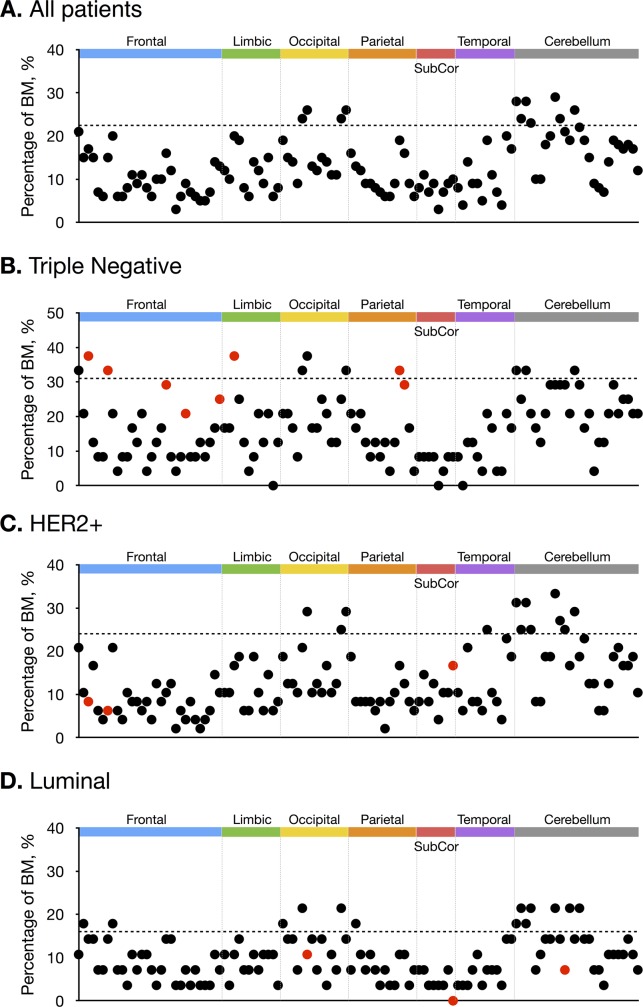
**The percentages of BMs were plotted across all patients (A), patients with triple negative gene type (B), patients with HER2+ gene type (C), and patients with Luminal type (D).** Brain regions were sorted in the order of the frontal, limbic, occipital, parietal, subcortical (SubCor), temporal, and cerebellum. Brain regions above dotted-line indicate the top 10% frequently occurring BMs. Reddish points indicate brain regions showing significant group differences from χ2 test.

Fig 2 and Fig 3 show frequency of occurrence of brain metastases in each subgroup. Top 10% regions in which metastases frequently occurred are evenly distributed in triple-negative, whereas BMs are concentrated in occipital, temporal lobe and cerebellum for HER2-positive, and in frontal, occipital lobe and cerebellum for luminal type.

### Comparison of distribution of brain metastases according to their subtypes (intergroup analysis)

BMs from triple-negative type breast cancer occurred more often in frontal lobe, limbic region, and parietal lobe, compared with other subtypes (corrected *P* < .05). BM from HER2-positive occurred less frequently in frontal lobe and subcortical region. BM from luminal type occurred less frequently in occipital lobe, subcortical region and cerebellum (corrected *P* < .05).

## Discussion

In this study, we evaluated the spatial distributions of BMs by breast cancer subtypes. We found that metastases from triple-negative breast cancer spread evenly in brain, but in case of HER2-positive and luminal types, BMs are concentrated in posterior circulation territories such as occipital lobe and cerebellum. In addition, compared with other subtypes, BMs from triple negative occur more frequently in frontal lobe, limbic region, and parietal lobe. The clinical implication of this observation is important because prophylactic irradiation with dose modulation to the preferential site is a viable approach for breast cancer to enhance its preventive effect and reduce any side effects.

Several studies have found that the cerebellum was the predominant site of metastases in breast cancer patients.[22–24] Our data also confirm that the cerebellum is the preferential site of breast cancer BMs. Conventionally, the “seed and soil theory” has been used to explain the preferential involvement of specific areas within the brain: the site of metastasis depends on the affinity of the tumor (the “seed”) to the microenvironment (the “soil”)[25]. Other potential explanations for preferential involvement of the cerebellum are as follows: (1) high gyral density of the cerebellar cortex compared to that of the cerebral hemispheres; (2) higher blood volumes and longer perfusion times of the tissue per minute in posterior circulation territories[26,27]; (3) different regional vasomotor response in the cerebellar circulation oriented toward a greater vessel dilatation[28,29].

However, recent studies have revealed the molecular mechanism of the spatial distribution of BMs. In breast cancer, chemokine receptors such as CXCR4 and CCR7 play a critical role in determining the metastatic destination of tumor cells[30]. COX2, EGFR ligand, and ST6GALNAC5 cross over the blood-brain barrier and enhance breast cancer metastasis to the brain. Evidence of specific subtypes showing a preference for brain metastasis is overwhelming. Triple-negative and HER2-positive breast cancer predispose to a higher risk of BM than that observed in luminal breast cancer, with an incidence of 30–40%[31–33]. A previous study reported that triple-negative type and HER2-positive breast cancer show an earlier onset of BMs than luminal breast cancer, which is consistent with our study[11,34]. In a recent study, WNT signaling was up-regulated in the triple-negative subtype and the BMs, but down-regulated in the luminal subtype and bone metastases[35]. Thus, we can assume that the spatial distribution of BMs differ according to the genetic composition of the primary breast cancer. Interestingly, our study showed that BMs of tripe-negative spread evenly in the whole brain, however HER-2 positive and luminal types have preferential involvements in the posterior circulation territories such as occipital lobe and cerebellum.

Despite neurosurgery and radiosurgery, triple-negative type breast cancer patients have the worst prognosis, with an overall survival duration of only 4.9 months.[36] Prophylactic cranial irradiation is a suitable treatment option for high-risk breast cancer. A randomized controlled trial showed that among 62 high-risk breast cancer patients receiving prophylactic cranial irradiation with 24 Gy in 10 fractions over 2 weeks, none developed BMs, but 6.4% of patients in the no prophylactic cranial irradiation arm developed BMs.[37] Thus, our results suggest different strategies of dose modulation of radiotherapy (whole brain coverage for triple-negative vs posterior circulation territories for HER2+ and luminal type).

Our results also may increase the diagnostic yield of brain metastases. Clinical information of triple-negative type of primary breast cancer could make radiologist to review, with a meticulous effort, occipital lobe and cerebellum as well as frontal, limbic and parietal lobe.

This study has a limitation. The number of cases was not enough to draw a solid conclusion. However, our results may serve as a cornerstone for future studies with a larger population to validate and extend these results.

In conclusion, different breast cancer subtypes may show different spatial distributions of BMs. Triple-negative breast cancer BMs has a tendency to spread evenly in the whole brain, meanwhile HER2+ and luminal types have a preponderance for posterior circulation territories. These results suggest different strategies for prophylactic irradiation according to subtypes (triple-negative vs other types).

## Supporting information

S1 File Statistics of brain metastases in ALL regions. (XLSX)
